# Nonsteroidal Anti-Inflammatory Drugs: A survey of practices and concerns of pediatric medical and surgical specialists and a summary of available safety data

**DOI:** 10.1186/1546-0096-8-7

**Published:** 2010-02-04

**Authors:** Deborah M Levy, Lisa F Imundo

**Affiliations:** 1 Division of Rheumatology, The Hospital for Sick Children and The University of Toronto, Toronto, Ontario, Canada; 2 Division of Rheumatology, Morgan Stanley Children's Hospital of New York-Presbyterian, Columbia University Medical Center, New York, NY, USA

## Abstract

**Objectives:**

To examine the prescribing habits of NSAIDs among pediatric medical and surgical practitioners, and to examine concerns and barriers to their use.

**Methods:**

A sample of 1289 pediatricians, pediatric rheumatologists, sports medicine physicians, pediatric surgeons and pediatric orthopedic surgeons in the United States and Canada were sent an email link to a 22-question web-based survey.

**Results:**

338 surveys (28%) were completed, 84 were undeliverable. Of all respondents, 164 (50%) had never prescribed a selective cyclooxygenase-2 (COX-2) NSAID. The most common reasons for ever prescribing an NSAID were musculoskeletal pain, soft-tissue injury, fever, arthritis, fracture, and headache. Compared to traditional NSAIDs, selective COX-2 NSAIDs were believed to be as safe (42%) or safer (24%); have equal (52%) to greater efficacy (20%) for pain; have equal (59%) to greater efficacy (15%) for inflammation; and have equal (39%) to improved (44%) tolerability. Pediatric rheumatologists reported significantly more frequent abdominal pain (81% vs. 23%), epistaxis (13% vs. 2%), easy bruising (64% vs. 8%), headaches (21% vs. 1%) and fatigue (12% vs. 1%) for traditional NSAIDs than for selective COX-2 NSAIDs. Prescribing habits of NSAIDs have changed since the voluntary withdrawal of rofecoxib and valdecoxib; 3% of pediatric rheumatologists reported giving fewer traditional NSAID prescriptions, and while 57% reported giving fewer selective COX-2 NSAIDs, 26% reported that they no longer prescribed these medications.

**Conclusions:**

Traditional and selective COX-2 NSAIDs were perceived as safe by pediatric specialists. The data were compared to the published pediatric safety literature.

## Introduction

Non-steroidal anti-inflammatory drugs (NSAIDs) are commonly prescribed for the symptomatic relief of pain and fever in children, and their anti-inflammatory effects are useful for juvenile arthritis and musculoskeletal (MSK) disorders. Traditional NSAIDs are non-selective cyclooxygenase (COX) inhibitors that inhibit both COX-1 and COX-2 enzymes. Inhibition of COX-1 dependent prostanoids disrupts cytoprotection of the stomach and platelet aggregation; therefore, blocking the COX-1 enzyme may result in abdominal pain, gastric irritation and gastrointestinal ulceration[1]. Inhibition of COX-2 dependent prostanoids, however, mediates anti-inflammatory, analgesic and antipyretic effects, and selective COX-2 inhibitors have diminished the gastrointestinal toxicities associated with NSAIDs[2,3]. Unfortunately, large randomized clinical trials of selective COX-2 inhibitors in adults detected an increased incidence of adverse cardiovascular events[3-6]. Further analysis demonstrated a similar cardiovascular risk with selective COX-2 inhibitors and traditional NSAIDs,[7] this risk likely due to permanent blockade of COX-2 dependent prostaglandins[8].

Unlike adults, gastrointestinal side effects, especially gastric ulcers, are infrequent in the pediatric population[9-11]. However, many children taking NSAIDs do report abdominal pain, nausea and anorexia[11,12]. In this population, a gastroprotective agent such as misoprostol, or a histamine (H2) blocker or proton-pump inhibitor along with the NSAID may be helpful[12-14], or for others a selective COX-2 NSAID may be an option.

NSAIDs are often prescribed "off-label" in pediatrics, although eight NSAIDs (including aspirin) are approved by the Food and Drug Administration (FDA) with indications for fever, pain or juvenile arthritis[15]. Two other NSAIDs have pediatric indications (indomethacin, for patent ductus arteriosus (PDA), and ketorolac administered in a single dose for pain). There are few studies of selective COX-2 NSAIDs in children, but include the treatment of acute post-surgical pain[16,17], juvenile arthritis[18,19], the arthropathy of hemophilia[20-22], and in the therapeutic regimen for fibrodysplasia ossificans progressiva[23]. Overexpression of the COX-2 enzyme has been recognized in childhood brain tumors, and may be a future target for treatment[24].

Despite the ubiquitous use of NSAIDs by physicians for pediatric patients, little has been published addressing their safety and tolerability. Limited pharmacokinetic studies of celecoxib[25], etodolac[26], and piroxicam[27] in children indicate significant differences which necessitate altered dosing.

The primary objectives of this study were to survey pediatric physicians who prescribe NSAIDs (traditional and selective COX-2) in order to describe the perceived efficacy, side effect profiles and adverse events attributable to this class of drugs. A secondary goal was to compare physician experience and perceptions to the published safety literature.

## Materials and methods

### Study Participants

An email inviting physicians to complete a web-based survey about NSAIDs was sent to 1289 physicians in the United States (US) and Canada. Random samples of physician emails were generated from membership rosters of the American Academy of Pediatrics (AAP), the American Pediatric Surgical Association, and the Pediatric Orthopaedic Society of North America. All members of the Sports Medicine Section of the AAP, the Pediatric Section of the American College of Rheumatology (ACR) and physician members of the Childhood Arthritis and Rheumatology Research Alliance (CARRA) were included. Individuals who appeared on more than one list were sent only one email. This method of compilation of names resulted in an intentional overrepresentation of pediatric rheumatologists and pediatric sports medicine physicians, two groups of physicians the investigators believed were frequent prescribers of NSAIDs. Initial email invitations were sent in May 2005 and two reminder emails were sent to non-responders. All surveys completed within 8 weeks were tabulated. Follow-up email requests were then sent to all respondents who indicated that they had observed a cardiovascular event in one or more patients. The study was approved by the Institutional Review Board at the Columbia University Medical Center.

### Survey Content

Questions were developed to elicit demographic information, prescribing practices of NSAIDs, physician perceptions of the frequency and occurrence of NSAID side effects, patient perceptions of NSAIDs, and attitudes and observations prior to, and following the withdrawal of rofecoxib (September 2004) and valdecoxib (April 2005). Questions were refined after pilot testing, and the final survey (available on request) was 22 questions or fewer, depending on the responses given, requiring 10 to 15 minutes to complete. Several questions asked physicians to determine the frequency of a response as "frequently" (more than once per week), "occasionally" (more than once per month), "rarely" (more than once per year) or "never". These questions addressed the frequency of prescribing specific drugs, prescribing for specific indications, observed side effects, and barriers to prescribing. All data were tabulated and compiled at the Center for Education Research and Evaluation, Columbia University; only de-identified data were provided to the investigators.

### Statistical Analysis

Percentages and frequencies were summarized to report the proportions of physician groups with different responses. Where appropriate, comparisons among groups were performed using chi-square, Fisher's exact test and Stuart-Maxwell tests of marginal homogeneity for paired categorical data. Statistical significance was defined as P < .05, using Stata 9.0 (College Station, TX) for analyses.

## Results

### Survey Respondents

1289 emails were sent and 84 were returned because of undeliverable email addresses. Three hundred and thirty-eight (28%) surveys were completed. Two respondents (one rheumatologist, one orthopedic surgeon) did not treat pediatric patients (<18 years), and six physicians have never prescribed an NSAID for a pediatric patient. Demographic data were collected from these surveys.

More than half of respondents were female (56%), and 52% were employed at a university teaching institution. Physician years in practice were evenly distributed (4% less than one year, 24% in practice 1 - 5 years, 18% in practice 6 - 10 years, 27% in practice 11 - 20 years, 23% in practice 21 - 30 years, and 6% in practice more than 30 years). Response rates varied by specialty; 168/635 (27%) AAP members ("pediatricians"), 100/247 (40%) pediatric rheumatologists, 12/106 (11%) sports medicine specialists, 24/145 (17%) pediatric surgeons, and 43/156 (28%) pediatric orthopedic surgeons responded. The total number of respondents was greater than 338 because several physicians identified themselves as belonging to more than one group. For analysis, data from the sports medicine subset were integrated into the pediatricians subset because only three respondents reported exclusively practicing sports medicine.

Data from 330 surveys were analyzed (those respondents who had ever prescribed NSAIDs to a patient less than 18 years old). Of the 330 physicians, 164 (49.6%) had never prescribed a selective COX-2 NSAID. By specialty, 72% (119/165) of pediatricians, 52% (22/42) of orthopedic surgeons, 79% (19/24) of pediatric surgeons, and 4% (4/99) of rheumatologists had never prescribed a selective COX-2 NSAID.

### Clinical Indications for NSAIDs

Traditional NSAIDs were prescribed for a myriad of reasons. Among all respondents, the most common reasons for ever prescribing a traditional NSAID were musculoskeletal (MSK) pain (91%), soft-tissue injury (82%), fever (80%), arthritis (78%), and headache (72%). For selective COX-2 NSAIDs, the most common reasons for ever prescribing were arthritis (78%), MSK pain (61%), soft tissue injury (37%), and fracture (23%). Indications for prescribing traditional and selective COX-2 NSAIDs for pediatricians and pediatric rheumatologists were similar (data not shown), except that a greater percentage of pediatric rheumatologists prescribed NSAIDs for juvenile arthritis (49% of pediatricians versus 100% of rheumatologists). Selective COX-2 NSAIDs were primarily prescribed after failure (lack of clinical efficacy or unacceptable side effects) of one or more traditional NSAIDs.

### Side Effects of NSAIDs

Respondents were asked to quantify the frequency of several common and rare side effects of NSAIDs. Because few side effects overall were reported by non-rheumatologists in all categories these data are not shown. Table 1 lists side effects reported by pediatric rheumatologists for traditional and selective COX-2 NSAIDs. Significantly more frequent reports of several side effects were noted for traditional NSAIDs compared to selective COX-2 NSAIDs.

**Table 1:** "Occasional" and "Frequent" NSAID Side Effects Reported by Rheumatologists

Side Effect number reported, (% of total)	Traditional (n = 99)	COX-2 (n = 95)	p-value
**Abdominal Pain**	80 (81)	22 (23)	< .001

**Gastric/duodenal ulcer**	6 (6)	2 (2)	0.28

**Hematuria**	4 (4)	1 (1)	0.37

**Hypertension**	3 (3)	3 (3)	1.0

**Thrombocytopenia**	1 (1)	0 (0)	1.0

**Epistaxis**	13 (13)	2 (2)	.006

**Easy Bruising**	63 (64)	8 (8)	< .001

**Headache**	21 (21)	1 (1)	< .001

**Fatigue**	12 (12)	1 (1)	.003

"occasional" = reported more than once per month, "frequent" = reported more than once per week, COX = cyclooxygenase. Only pediatric rheumatologists who prescribed these drugs were included, therefore, the sample sizes of the comparison groups were different. Statistical comparisons were made by Fisher's exact test.

### Adverse Cardiovascular Effects

To assess cardiovascular side effects of NSAIDs, questions asked specifically about stroke, myocardial infarction, angina, congestive heart failure and unexplained cardiac death. Eleven physicians reported that one or more of their patients had had an event, and these respondents were contacted (with a 100% response rate) to obtain further details. The cardiovascular events were attributed to underlying diagnoses of systemic lupus erythematosus or antiphospholipid syndrome (stroke), sickle cell anemia (congestive heart failure, stroke), hypertension (stroke) or congenital heart disease (stroke, myocardial infarction and congestive heart failure). No events were attributed by the physician to the use of either a traditional or selective COX-2 NSAID. No unexplained cardiac deaths were reported.

### Selective COX-2 NSAIDs

**Figure 1:**
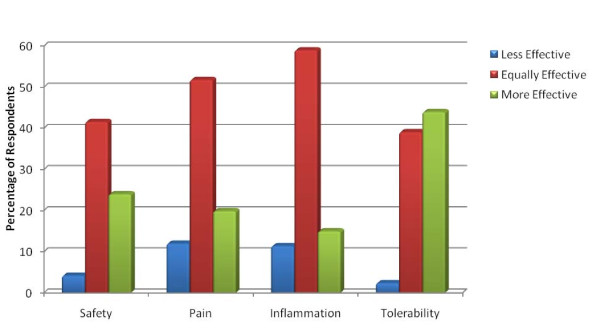
**COX-2 versus Traditional NSAIDs: Physician Perceived Differences are Minimal Except for Tolerability**. Respondents compared selective COX-2 NSAIDs to traditional nonselective NSAIDs for their safety, pain relief, anti-inflammatory effects and tolerability; available choices were "less effective", "equally effective", "more effective", or "cannot give an opinion" (not shown).

To compare traditional NSAIDs to selective COX-2 NSAIDs, questions were directed only to physicians who had ever prescribed a COX-2 NSAID (166/330); results are grouped for all respondents in Figure 1. Respondents rated COX-2 NSAIDs as equivalent or superior to traditional NSAIDs for safety (66%), pain relief (72%), relief of inflammation (74%) and tolerability (83%).

**Figure 2:**
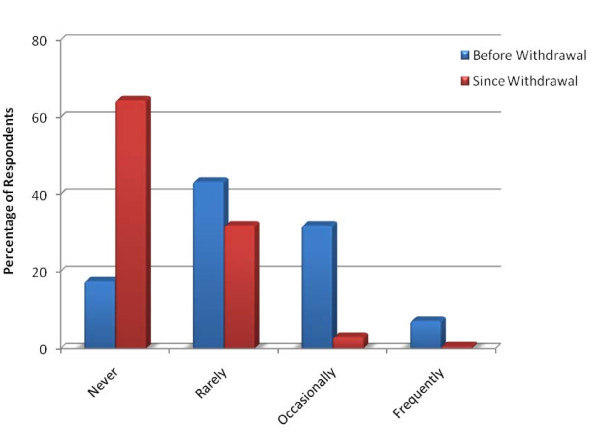
Patient/Caregiver requests for selective COX-2 NSAIDs have decreased since the voluntary withdrawal of rofecoxib and valdecoxib*. *These distributions are significantly different (p < 0.001) by chi-squared analysis with Stuart-Maxwell test of homogeneity. Rarely = more than once per year, occasionally = more than once per month, frequently = more than once per week.

Attitude changes of patients and/or their caregivers (as expressed to their physician) prior to, and following, the voluntary withdrawal of rofecoxib and valdecoxib were explored. Figure 2 illustrates that specific patient and/or caregiver requests for selective COX-2 NSAIDs decreased in frequency (p < .001) following the drug withdrawals. Similarly, physician prescribing habits changed after the drug withdrawals. Of the pediatric rheumatologists who responded, 44% reported an increased number of prescriptions of traditional NSAIDs, 53% reported no change in prescribing habits, and 3% reported giving fewer traditional NSAID prescriptions. For selective COX-2 NSAIDs, however, less than 1% of pediatric rheumatologists increased prescribing, 57% gave fewer prescriptions, and 26% reported that they no longer prescribed these medications.

**Figure 3:**
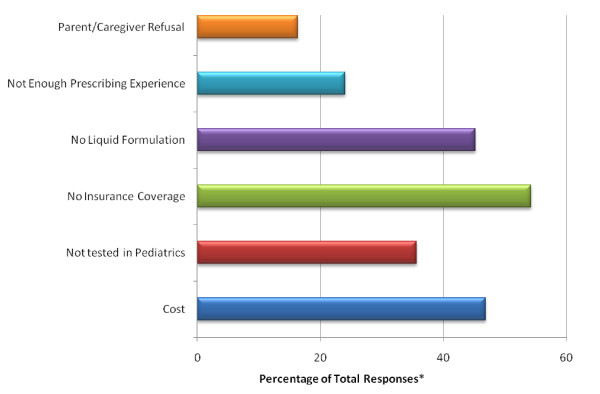
**Barriers to Prescribing Selective COX-2 NSAIDs**. *Percentage of all responses that were either "occasionally" (more than once per month) or "frequently" (more than once per week).

Barriers to prescribing selective COX-2 NSAIDs for pediatric patients were examined; at the time of this survey, no drug in this class had a pediatric indication. Cost, lack of testing in children, lack of insurance coverage and no available liquid formulation were reported as barriers "occasionally" or "frequently" by at least 25% of respondents who prescribed these drugs (Figure 3).

### Prescribing Habits by Specialty

Physicians were asked how often they prescribed each of the available NSAIDs (not just those with pediatric indications). Non-rheumatologists "frequently" (more than once per week) prescribed ibuprofen, naproxen, and ketorolac, and "rarely" (less than once per year) prescribed any other drug in this class. Eighty percent of pediatric surgeons and 52% of orthopedic surgeons prescribed ketorolac "occasionally" (more than once per month) or "frequently". Pediatric rheumatologists prescribed a wider range of drugs, but only ibuprofen, diclofenac, indomethacin, naproxen, celecoxib and rofecoxib were prescribed by at least 40% of the sample.

## Discussion

This study is the first to examine pediatric physicians' perceptions of the safety and tolerability of NSAIDs as prescribed for their pediatric patients. Overall, physicians who responded to this survey regard NSAIDs - both traditional and selective COX-2 - as safe for use in the pediatric population. NSAIDs were frequently prescribed for musculoskeletal and soft tissue pain, followed by fever, headaches, and juvenile arthritis. The most commonly observed side effects were abdominal pain, epistaxis, bruising, headache and fatigue. Significantly fewer prescriptions of selective COX-2 NSAIDs were written since the voluntary withdrawal of two drugs in this class, although observed side effects were reported significantly less frequently among children who received these medicines. Although the survey data are subjective, little published safety data of selective COX-2 NSAID use in children exists. Facing a lack of available data, the prescribing of and taking of all NSAIDs has recently come under close scrutiny.

### Survey Bias

This survey was distributed to a large number of physicians, with a 28% completion rate; lower than the average mail survey response rate[28], but consistent with literature that suggests a lower response rate for web-based surveys[29-31]. Therefore, in consideration of possible bias, and thus the validity of the study, it is likely that the data are skewed toward over-reporting of NSAID side effects. Presumably, physicians who followed the reporting of NSAID concerns developed opinions (either positive or negative), and were more likely to respond to the introductory email explaining the objectives. Respondents were also more likely interested in how NSAIDs are prescribed and by whom they are taken. There were many reasons that physicians chose not to respond, specific comments included "the overabundance of surveys received", while a lack of time and energy, lack of interest or personal applicability to the subject matter, and a general reluctance to participate in another's research were reported by nonparticipants. These reasons are consistent with the recent medical survey literature[32,33]. Many of the recommended strategies to improve response rates were employed, including sending reminders, using email as a communication tool, offering minimal incentives (lottery tickets), and personalizing the introductory emails[30,31,34].

This study was skewed to include a large percentage of pediatric rheumatologists, as the investigators were particularly interested in physician groups with extensive experience prescribing NSAIDs. Survey responses were based on subjective pooling of data - i.e. physicians were asked to estimate how often they observed specific side effects, or how often they prescribed specific drugs. Respondents were not asked to recall specific patients or to provide numerical frequencies of prescribing, thus, the data were possibly biased toward either over- or underreporting, depending on the actual frequency of the side effect observed in practice - common side effects may have been underreported and rare side effects overreported.

### Prescribing Habits and Barriers

Despite the study limitations, the data confirms that pediatricians and pediatric subspecialists continue to prescribe NSAIDs (98% of respondents have prescribed an NSAID for a pediatric patient), but that known side effects influence their choice of drugs. Selective COX-2 NSAIDs have been prescribed by just over half of all respondents, suggesting either a decreased perception of need, or recent apprehension of these medications. Factors for not prescribing specific NSAIDs included a perceived lack of efficacy, perceived danger by either physicians or patients, lack of testing in pediatric populations, greater expense, lack of appropriate (liquid) formulation, and lack of an FDA-approved pediatric indication. Many physicians also expressed unfamiliarity with the specific COX-2 NSAIDs.

The survey asked physicians to consider only their NSAID prescribing habits, thus, it did not capture data about general prescribing habits for disease, nor did it attempt to quantify "co-prescribing", for example, the number of patients who received other drugs such as methotrexate or a biologic response modifier, in addition to an NSAID, for the treatment of juvenile arthritis.

### Safety of NSAIDs in Children

Side effects of NSAIDs are common, but severe side effects are rare in children. A review of the safety literature of NSAID use in pediatrics revealed controlled studies of ibuprofen, ketorolac, naproxen, meloxicam, rofecoxib and celecoxib. The largest randomized trial of ibuprofen (versus acetaminophen) included more than 84,000 children with fever. The observed risk of gastrointestinal bleeding in the ibuprofen group was 7.2 per 100,000, not significantly different from the comparison group[35]. An older study demonstrated a dose-response relationship with ibuprofen and adverse gastrointestinal reactions[36]. A recent Cochrane Review concluded that ibuprofen should not be used prophylactically for PDA closure because of potential side effects including decreased urine output, increased creatinine, and pulmonary hypertension[37].

Studies of ketorolac to determine its safety for neonates after cardiac surgery have demonstrated no significant increase in wound or gastrointestinal bleeding, or changes in renal function[38-40]. In older children, no significant increased incidence of bleeding was observed in three separate studies[41-43]; however, one observational study demonstrated two potentially associated bleeding events[44]. The current study confirms that ketorolac was prescribed frequently by surgeons and non-surgical specialists alike, without reported bleeding side effects.

A randomized, double-blind trial of meloxicam versus naproxen for juvenile arthritis reported gastrointestinal symptoms in up to 38% of subjects, and other adverse events including headache and rash in fewer than 15%. No ulcerations, perforations or major bleeds were reported although "bleeding disorders" (rectal, epistaxis, hematuria, hematoma, purpura) were reported in 6.2%[45]. Another study reported adverse events (abdominal pain, diarrhea, nausea and increased hepatic enzymes or blood urea nitrogen) in 11% of patients believed to be related to the drug[46]. In this survey, meloxicam was prescribed predominantly by pediatric rheumatologists, probably due to the lack of studies for any other indication.

Two randomized double-blind, placebo controlled trials of rofecoxib in children undergoing adenotonsillectomy noted no increased bleeding due to rofecoxib[16,17]. A randomized double-blind, double-dummy study of rofecoxib versus naproxen for juvenile arthritis reported abdominal pain in up to 39% of subjects in each group (no significant difference). Headache, rash, pharyngitis and upper respiratory infections were observed in fewer than 15%, and two cases of edema were deemed possibly or probably related to the drug[19].

The pediatric literature of non-controlled studies of NSAID safety includes reports of reversible renal failure and significant gastropathy[47-51]. In this survey, adverse renal events were rarely reported, probably an underreporting bias since questions addressed only hematuria and hypertension, and did not ask about episodes of renal failure. The largest study of gastropathy in children taking NSAIDs retrospectively reviewed 702 patients with juvenile arthritis; five children with 10 gastropathy events were documented by barium swallow or endoscopy and thought to be attributable to NSAIDs[9]. Gastropathy may occur more frequently, as another study of 14 children with juvenile arthritis taking NSAIDs for at least one year demonstrated endoscopic lesions in 43%, abdominal pain in 27% and H. pylori in 57%[52]. Recently, four children given ibuprofen for fever developed isolated gastric antral ulcers following the first or second dose[10]. The current survey was not designed to ascertain the incidence of gastropathy; however, physicians reported abdominal pain as a frequent side effect, and ulceration as a rare side effect.

### Tolerability of NSAIDs in Children

This survey demonstrated a lower frequency of several observed side effects when selective COX-2 NSAIDs were prescribed compared to traditional NSAIDs. Eighty percent of physicians reported observing "occasional" to "frequent" abdominal pain for traditional NSAIDs, compared to 23% for selective COX-2 NSAIDs (p < .001). This finding may be biased, as patients with abdominal pain were more likely to receive selective COX-2 NSAIDs with the expectation that their abdominal pain would resolve. Other significant differences were observed in the frequency of headaches, epistaxis, fatigue and easy bruising. As the duration and breadth of experience with selective COX-2 NSAIDs is less (fewer patient years of experience), observed side effects would be unusual unless they commonly occur. This is true of the adult literature, where cardiovascular side effects of selective COX-2 NSAIDs were only noted after thousands of patients were studied[7]. Collectively, pediatric rheumatologists have thousands of patient years of experience prescribing traditional NSAIDs, and perhaps only hundreds with the selective COX-2 NSAIDs, thereby accounting for fewer observed, or perceived side effects.

### Cardiovascular Side Effects of NSAIDs

In this sample of 338 physicians, eleven reported one or more adverse cardiovascular events in patients taking NSAIDs. All of the events were attributed to an underlying medical diagnosis (complex congenital heart disease, systemic lupus erythematosus, sickle cell anemia, or antiphospholipid syndrome), and none of these events were attributed to NSAID use. Thus, both traditional and selective COX-2 NSAIDs were perceived as safe for pediatric patients.

## Conclusions

At present, there are nine traditional NSAIDs (aspirin, etodolac, ibuprofen, indomethacin, ketorolac, meloxicam, naproxen, oxaprozin, and tolmetin) and one selective COX-2 NSAID (celecoxib) with FDA-approved pediatric indications. Thus study suggests that pediatricians are comfortable prescribing the traditional NSAIDs, but few practitioners aside from rheumatologists prescribe selective COX-2 NSAIDs. Recent publications and media attention have altered attitudes towards NSAID prescribing. The published safety data of these drugs in pediatrics are limited, although few serious adverse events have been reported. Pediatric pharmacokinetics and risk factors differ from adults, thus phase IV, open-label post-marketing studies of these drugs as prescribed for children must be initiated in order to accurately assess the potential risks of this valuable class of medications. Otherwise, physician and patient perceptions of these drugs will continue to bias clinical practice.

## List of Abbreviations

AAP: American Academy of Pediatrics; ACR: American College of Rheumatology; CARRA: Children's Arthritis and Rheumatology Research Alliance; COX: cyclooxygenase; FDA: Food and Drug Administration; MSK: musculoskeletal; NSAID: nonsteroidal anti-inflammatory drug; PDA: patent ductus arteriosus.

## Competing interests

DML was supported by an independent research grant from Pfizer, Inc. to support this study. LFI has no competing interests.

## Authors' contributions

DL designed the study, performed the statistical analysis and drafted the manuscript. LI participated in the design of the study and helped to draft the manuscript. All authors read and approved the final manuscript.
